# Awareness, knowledge and attitude toward influenza vaccination in several population groups in China: A cross-sectional study

**DOI:** 10.3389/fpubh.2022.950532

**Published:** 2022-10-13

**Authors:** Binshan Jiang, Zhenzhong Wang, Mengmeng Jia, Huijiao Yan, Zheng Su, Shujun Liu, Weizhong Yang, You-lin Qiao, Luzhao Feng

**Affiliations:** ^1^School of Population Medicine and Public Health, Chinese Academy of Medical Sciences and Peking Union Medical College, Beijing, China; ^2^National Cancer Center/National Clinical Research Center for Cancer/Cancer Hospital, Chinese Academy of Medical Sciences and Peking Union Medical College, Beijing, China

**Keywords:** influenza vaccine, awareness, knowledge, vaccination willingness, recommendation willingness

## Abstract

**Background:**

We aimed to comprehensively analyze awareness, knowledge and attitude toward influenza vaccine and the factors associated to vaccine acceptance among the young and middle-aged general population, healthcare workers, and health-related administrators in China. The factors influencing the promotion of influenza vaccination were also evaluated among healthcare workers and administrators.

**Methods:**

This is a multicenter, cross-sectional study. General population adults, healthcare workers (HCWs), and health administrators were enrolled in seven regions across China during the 2020–2021 flu season. Data were collected *via* an online questionnaire, which included information request as to awareness, knowledge, and attitude toward influenza vaccination. Statistical significance set at *p*-values < 0.05.

**Results:**

A total of 3,239 individuals were included in our analyses. There were gaps in consciousness to action, especially between awareness (87.1%) and knowledge (57.7%), and between willingness (57.3%) and vaccination (22.3%). The downward trends were similar in all three groups. HCW group and the health administrator group showed more positive propensity to accept influenza vaccines than the general population group. For the general population group, those with a lower educational level (lower than a bachelor's degree) were less likely to be vaccinated (aOR = 0.66, 95% CI: 0.45–0.96). For the HCW group, practitioners older than 45 years were more reluctant to be vaccinated than those under 25 years (aOR = 0.41, 95% CI: 0.19–0.86). For the health administrator group, personnel aged 26 years and above were less inclined to be vaccinated (aORs = 0.17–0.20). In all groups, people who had received influenza vaccines in the past 5 years (aOR = 1.72, 95% CI: 1.31–2.26 in general population group, 13.05, 95% CI: 7.71–22.10 in HCW group, and 19.30, 95% CI: 9.66–42.63 in health administrator group) were more likely to be vaccinated in future seasons. People who were not covered by the free program or those without awareness of the related programs were less likely to be vaccinated (aORs < 0.63). Most (70.8%) of HCWs showed intention to recommend the influenza vaccine. Clinical doctors, those who had flu shots themselves, and those who had more knowledge, were more like to make recommendations. Health administrators stated that insufficient budget resources and workforce, and low public awareness are main difficulties in the promotion of influenza vaccine.

**Conclusion:**

The influencing factors of the attitude toward influenza vaccination vary across populations. Governments need to carry out focused vaccination promotion programs, especially for healthcare workers, to improve the coverage of influenza vaccination.

## Introduction

Historically, influenza viruses have caused substantial mortality associated with seasonal influenza (1). Approximately 20% of unvaccinated children and 10% of unvaccinated adults are infected every year (2). It is estimated that there were 3 million influenza-related cases of influenza-like illness in out-patient and emergency departments, 2.34 million in-patient cases of severe acute respiratory infection, and 88 thousand respiratory deaths on average every year between 2006 and 2019, before Coronavirus disease 2019 (COVID-19) pandemic in China (3).

Influenza vaccination is recognized as the most cost-effective way to prevent seasonal influenza infection and its potentially deadly complications, and annual vaccination is recommended by World Health Organization, Advisory Committee on Immunization Practices, and the Technical Working Group on National Immunization Programmes Influenza Vaccine Working Group in China (3). The number of influenza vaccine doses given in China had gradually increased to 57.7 million shots in 2020 since it hit a record low in 2018, however, in 2021 there was a sharp drop of 96%, to <2.2 million (4). The seasonal influenza vaccination rate was discouraging for a country with 1.4 billion people, which achieved only 15% among healthcare workers (HCWs) (5). The non-pharmaceutical interventions (NPIs) taken to contain COVID-19 pandemic played a positive role in preventing and controlling the spread of seasonal influenza and other respiratory infections (6), which resulted in a 94% reduction in incidence in the month following the implementation of strict NPIs (7, 8). However, scientists are concerned that the unremitting flatness of seasonal influenza activity trends and undesirable vaccine uptake rate may lower the public's immunity acquired by natural infection or immunization, especially for children and elderly at high risk (9, 10). Influenza vaccination is critical for preventing the rebound of an influenza epidemic, as NPIs are relaxed (11, 12).

In countries with large population densities like China, influenza transmission is common among household contacts, from infected individuals to vulnerable household members (13, 14). Those young or middle-aged who have mild or no symptoms, are likely to be the infection source in the family and community. Given that most young-middle aged individuals are employed, the influenza infection may also cause absenteeism or influenza spread within the workplace, it is also important to evaluate the willingness to get the influenza vaccination among the middle-aged general population. Furthermore, those that work in hospitals are a priority for influenza vaccination with a higher risk of occupational exposure and playing a critical role in disrupting influenza transmission (15). In addition, their attitude toward the influenza vaccine also affects their patients (16, 17). The promotion of influenza vaccination is also affected by health-related administrators and policies (18). The fact that COVID-19 vaccines can prevent severe and fatal cases has enhanced public confidence in vaccines (19), making it an opportune time to maintain the increasing flu vaccination rates as a more powerful strategy than limiting social contacts (20).

Thus, we accessed the public's willingness to receive the influenza vaccine and hoped that the findings would provide a reference to improve immunization in China. As a joint effort of multiple sectors, we comprehensively analyzed the awareness, knowledge and attitude toward the vaccine in the young and middle-aged general population, HCWs, and health-related administrators separately. This survey also evaluated the factors influencing the promotion of influenza vaccination among HCWs and administrators.

## Methods

### Study design and population

This multicenter, cross-sectional study was conducted in mainland China during the 2020–2021 season between December 2020 and April 2021. Convenient sampling methods were used throughout seven geographically administrative regions of China to enroll participants above 18 years old. Those without signs of cognitive impairment and agree to participate in this survey were eligible. The participants in general population group were recruited from the community. HCW group refers specific to doctors and nurses recruited from hospitals, which administrative and research staff were excluded. Health administrator group refers to staff taking office at health commissions, Centers for Disease Prevention and Control, community healthcare centers, and hospitals in charge of vaccination. The face-to-face survey was carried out by trained investigators with self-designed questionnaire. All data were collected with an online platform (Wenjuanxing, Changsha Haoxing Information Technology Co. Ltd., Changsha, China) during or after the interviews.

### Sample size

The sample size per group was estimated by the formula $N=\mu_{\alpha }^{2}\times p\times \;\left(1-p\right)/\delta^{2}$, based on 5% type one error, the rate of willingness of influenza vaccine in the general population group (p) = 50%, and maximum permissible error (δ) = 0.1p. We estimated a sample size of 385 participants each group. Considering the potential invalid response, the sample size was 424 per group after increased 10%. Given that there are seven geographically administrative regions across China, each region was assigned at least 61 participants per group.

### Measurement

Sociodemographic characteristics of participants, including age, gender, living area, ethnicity, educational background, marital status, annual household income, were collected. Their influenza vaccination history and attitude were also collected.

The primary outcome variables were the awareness, knowledge, and favorable willingness rates toward the influenza vaccine in the 2020–2021 season. Awareness and knowledge of the influenza vaccine were determined using the question: “Do you know about the influenza vaccine? (No/Yes, but do not know the use of it/Yes, it can prevent all colds/Yes, it can only prevent flu)”. The awareness of the influenza vaccine was defined as respondents knowing that influenza vaccines have already existed. Among them, only those who had correct information about it were regarded to equip knowledge of the influenza vaccine. Thus, the awareness rate was computed with answers including “Yes”, while the knowledge rate was determined by the answer “Yes, it can only prevent flu.” Willingness to be vaccinated was defined using the question: “Are you going to get influenza vaccines this season if available? (Strongly willing/Willing/Not sure/Unwilling/Strongly unwilling)” The willingness rate was computed with the answers including “strongly willing” and “willing”, others were categorized as hesitancy. The knowledge of seasonal influenza was asked with the question: “Do you think influenza is the same as a regular cold? (Yes/No/Unclear)”.

The secondary outcome variables were the recommendation of influenza vaccination by HCWs, and the promotion preference by administrators. The recommendation intention of HCWs was measured by the question: “Would you recommend influenza vaccination for those without contraindications? (Yes/Neutral/No)”. Answers “neutral” and “no” were merged into one group during analyses. The preference of health administrator group on promoting influenza vaccination was evaluated by the question: “Do you agree that governments should take charge of influenza vaccination promotion? (Yes/No)”. Multiple choice questions were offered to identify the reasons of their choice, and their suggestions toward influenza vaccination delivery.

### Statistical analysis

Data were exported into Microsoft Excel and automatically filled by the Wenjuanxing online questionnaire administration system. Data were analyzed with SPSS version 24 (Armonk, NY, USA) and R version 4.1.3 (R Foundation for Statistical Computing, Vienna, Austria). Absolute and relative frequencies were calculated for categorical variables, and Chi-square test was used for comparison. Associations between sociodemographic factors (age groups, gender, ethnicity, educational level, marital status, place of residence, and self-reported financial status) and influenza vaccination (awareness, knowledge and positive willingness) were analyzed using the multivariate logistic regression analyses. Predictors of intention to recommendation were analyzed by both univariate and multivariate logistic regression. Results are presented with adjusted odds ratios (aORs) and 95% confidence intervals (CIs). The level of statistical significance was a *p*-value < 0.05 (two-tailed). Bar graphs were used to illustrate the cascade of influenza vaccination behavior, and an additional Sankey diagram to visualize this evolution from consciousness to action progress.

### Ethics

This study was reviewed and approved by the National Cancer Center/Cancer Hospital, Chinese Academy of Medical Sciences and Peking Union Medical College (approval number: 20/054-2250, approval date: March 12, 2020). Informed consent was obtained at the beginning of the survey, and those who failed to provide consent could not have access to the survey. Datasets were anonymous and prevented the identification of any individual study subject by the research team at any stage of the study.

## Results

### Demographic characteristics and influenza vaccination behaviors

A total of 3,547 questionnaires were collected in this survey with a response rate of 97.7%, of which 3,239 (91.3%) valid records were included. Among the 3,239 respondents, 1,845 (57.0%) came from the general population group, 1,010 (31.2%) from the HCW group, and 384 (11.9%) from the health administrator group. The overall mean age was 32.0 (SD ± 10.3) years. As shown in Table 1, respondents were predominantly female (67.4%) and resided in urban areas (81.8%). Retrospective behavior more than 6 months in the past, 402 (12.4%) respondents reported smoking, and 508 (15.7%) were considered alcohol consumers status.

**Table 1:** Participant characteristics and their attitude toward influenza vaccines.

**Characteristics**	**Overall (*n* = 3,239)**	**General population (*n* = 1,845)**	**Healthcare workers (*n* = 1,010)**	**Health administrator (*n* = 384)**	***p*-value**
	*N* (%)^*^	*N* (%)^*^	*N* (%)^*^	*N* (%)^*^	
Age, years					
Mean (SD)	32.0 (10.3)	28.4 (9.8)	36.5 (8.7)	37.6 (9.0)	<0.001
18–25	1,133 (35.0)	1,035 (56.1)	80 (7.9)	18 (4.7)	<0.001
26–35	1,091 (33.7)	481 (26.1)	443 (43.9)	167 (43.5)	
36–45	564 (17.4)	147 (8.0)	300 (29.7)	117 (30.5)	
>45	451 (13.9)	182 (9.9)	187 (18.5)	82 (21.4)	
Gender					
Female	2,183 (67.4)	1,172 (63.5)	777 (76.9)	234 (60.9)	<0.001
Male	1,056 (32.6)	673 (36.5)	233 (23.1)	150 (39.1)	
Ethnic					
Han	2,903 (89.6)	1,656 (89.8)	890 (88.1)	357 (93.0)	0.028
Others	336 (10.4)	189 (10.2)	120 (11.9)	27 (7.0)	
Educational background					
Master and above	674 (20.8)	277 (15.0)	280 (27.7)	117 (30.5)	<0.001
Bachelor or equal	2,203 (68.0)	1,247 (67.6)	711 (70.4)	245 (63.8)	
Lower than bachelor	362 (11.2)	321 (17.4)	19 (1.9)	22 (5.7)	
Place of residence					
Urban	2,650 (81.8)	1,326 (71.9)	963 (95.3)	361 (94.0)	<0.001
Rural	589 (18.2)	519 (28.1)	47 (4.7)	23 (6.0)	
Region					
Northeast China	331 (10.2)	213 (11.5)	68 (6.7)	50 (13.0)	<0.001
Northern China	1,229 (37.9)	545 (29.5)	621 (61.5)	63 (16.4)	
Eastern China	603 (18.6)	459 (24.9)	72 (7.1)	72 (18.8)	
Southern China	248 (7.7)	152 (8.2)	50 (5.0)	46 (12.0)	
Central China	357 (11.0)	253 (13.7)	53 (5.2)	51 (13.3)	
Northwestern China	272 (8.4)	161 (8.7)	53 (5.2)	58 (15.1)	
Southwestern China	199 (6.1)	62 (3.4)	93 (9.2)	44 (11.5)	
Marital statue					
Unmarried	1,639 (50.6)	1,318 (71.4)	228 (22.6)	93 (24.2)	<0.001
In marriage	1,528 (47.2)	490 (26.6)	753 (74.6)	285 (74.2)	
Others^#^	72 (2.2)	37 (2.0)	29 (2.9)	6 (1.6)	
Annual household income, thousand yuan					
<50	779 (24.1)	603 (32.7)	133 (13.2)	43 (11.2)	<0.001
60–100	1,298 (40.1)	704 (38.2)	462 (45.7)	132 (34.4)	
110–350	1,044 (32.2)	462 (25.0)	389 (38.5)	193 (50.3)	
>350	118 (3.6)	76 (4.1)	26 (2.6)	16 (4.2)	
Smoking status					
Yes	402 (12.4)	266 (14.4)	91 (9.0)	45 (11.7)	<0.001
No	2,837 (87.6)	1,579 (85.6)	919 (91.0)	339 (88.3)	
Drinking alcohol status					
Yes	508 (15.7)	300 (16.3)	127 (12.6)	81 (21.1)	<0.001
No	2,731 (84.3)	1,545 (83.7)	883 (87.4)	303 (78.9)	
Flu vaccination history in previous 5 years					
Yes	721 (22.3)	297 (16.1)	243 (24.1)	181 (47.1)	<0.001
No	2,518 (77.7)	1,548 (83.9)	767 (75.9)	203 (52.9)	
Do you think influenza is the same as a regular cold?					
Yes	355 (11.0)	214 (11.6)	113 (11.2)	28 (7.3)	<0.001
No	2,402 (74.2)	1,262 (68.4)	810 (80.2)	330 (85.9)	
Unclear	482 (14.9)	369 (20.0)	87 (8.6)	26 (6.8)	
Have you heard of influenza vaccine?					
Yes	2,820 (87.1)	1,505 (81.6)	942 (93.3)	373 (97.1)	<0.001
No	419 (12.9)	340 (18.4)	68 (6.7)	11 (2.9)	
What do you know about the influenza vaccine?					
It can only prevent flu.	1,870 (57.7)	811 (44.0)	730 (72.3)	329 (85.7)	<0.001
It can prevent all colds.	346 (10.7)	215 (11.7)	109 (10.8)	22 (5.7)	
Just heard of it, but not know the use of it.	604 (18.6)	479 (26.0)	103 (10.2)	22 (5.7)	
Nothing	419 (12.9)	340 (18.4)	68 (6.7)	11 (2.9)	
Is there any preferential policy to vaccinate key groups against influenza in your residence?					
Yes	930 (28.7)	484 (26.2)	292 (28.9)	154 (40.1)	<0.001
No	823 (25.4)	412 (22.3)	285 (28.2)	126 (32.8)	
Unclear	1,486 (45.9)	949 (51.4)	433 (42.9)	104 (27.1)	
If yes, would you like to get the flu vaccination?					
Yes	1,924 (59.4)	1,016 (55.1)	625 (61.9)	283 (73.7)	<0.001
Unsure	1,039 (32.1)	650 (35.2)	306 (30.3)	83 (21.6)	
No	276 (8.5)	179 (9.7)	79 (7.8)	18 (4.7)	
Are you going to get influenza vaccines this season if available?					
Willing	1,856 (57.3)	980 (53.1)	605 (59.9)	271 (70.6)	<0.001
Hesitancy	1,383 (42.7)	865 (46.9)	405 (40.1)	113 (29.4)	

*Percentages may not total to 100 owing to rounding.

#Others included divorced and widowed.

Smoking was defined as continuous or accumulated smoking for more than 6 months in the past.

Drinking was defined as one or more times a week for six consecutive months, whether it was liquor, beer, wine, or yellow rice wine.

For question “Flu vaccination history in previous 5 years”, answer yes included self-reported having one or more times in the past 5 years.

**Figure 1:**
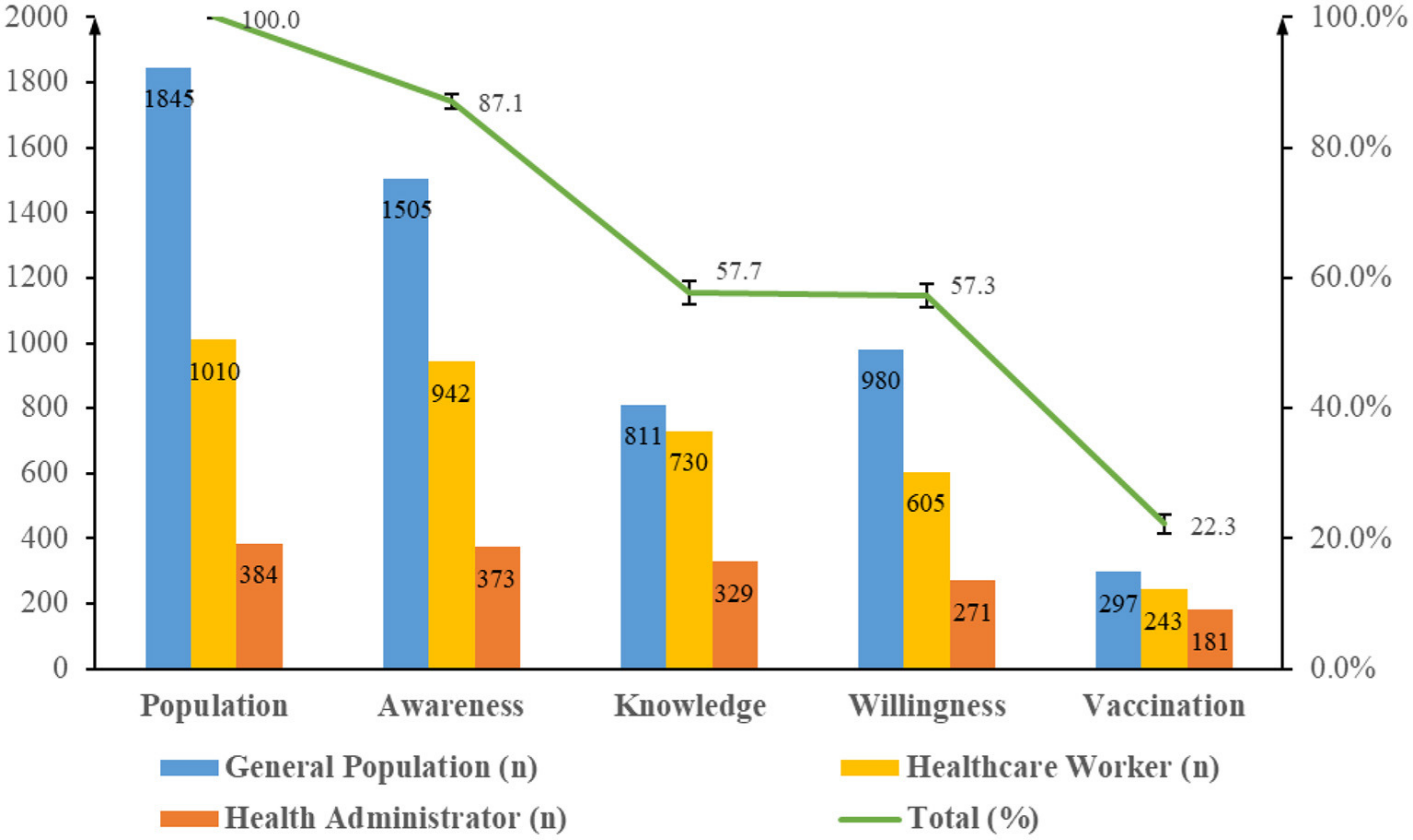
Gaps in awareness, knowledge, willingness, and vaccination behavior toward influenza vaccine. The numbers of awareness, knowledge, willingness, and 5-year vaccination rate among general population, healthcare worker, and health administrator groups was calculated and shown in the graph. Rates of awareness, knowledge, willingness, and 5-year vaccination rate in all respondents was calculated and shown by the broken line.

**Figure 2:**
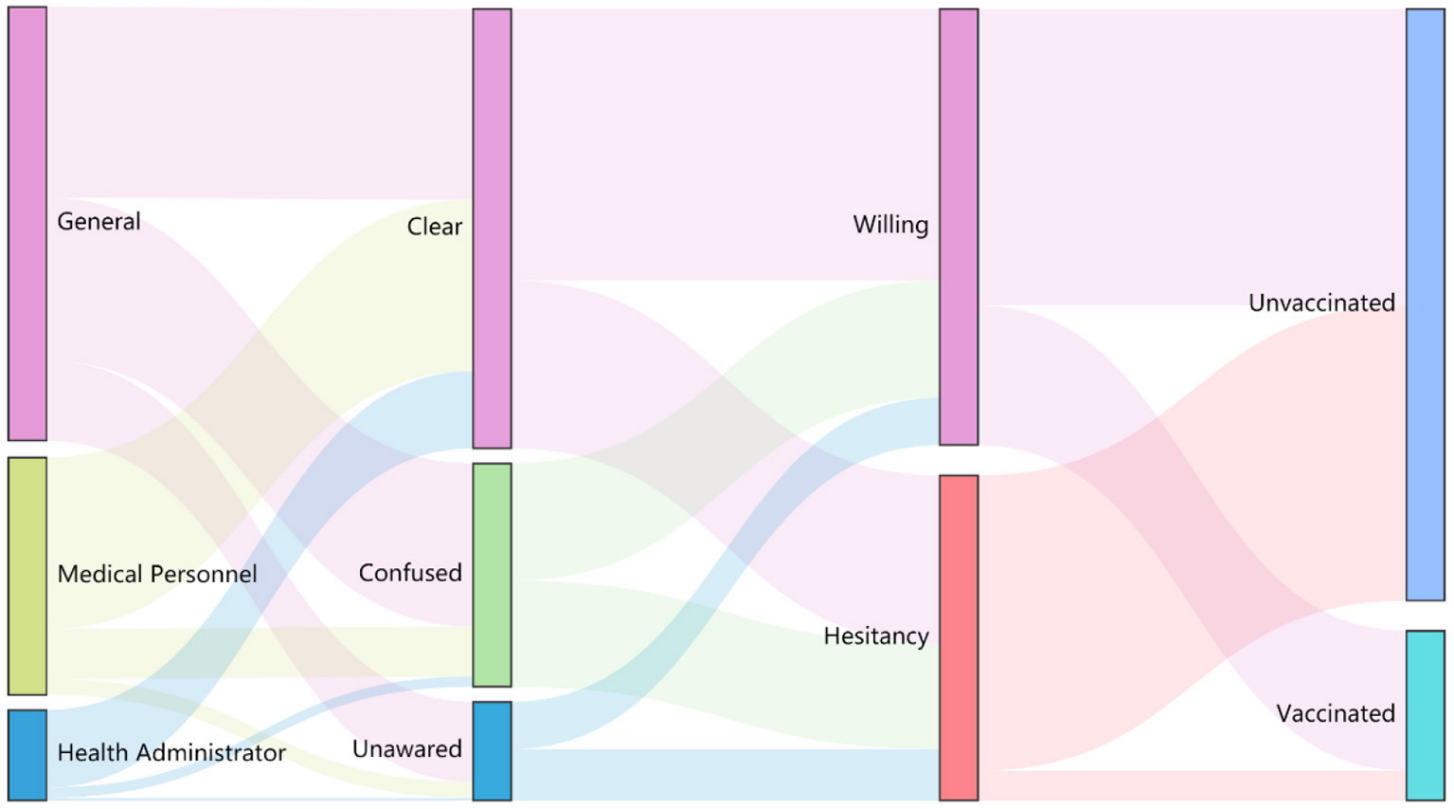
Behavior pattern Sankey diagram showed leading nodes of vaccination rates among groups.

There were gaps in consciousness to action, especially between awareness (87.1%) and knowledge (57.7%), and between willingness (57.3%) and vaccination uptake (22.3%). As the influenza vaccination behavior cascade shown in Figure 1, the downward trends of the three groups appeared to be similar, with one exception: willingness (53.1%) was higher than knowledge (44.0%) in the general population group. Although the groups' results of knowledge and willingness were close, they are not always the same participants, as shown by different color flows in the Sankey diagram (Figure 2).

### Awareness and knowledge of influenza vaccine

Most respondents (2,820/3,239, 87.1%) were aware of the influenza vaccine, including the health administrator group (97.1%), however, only 1,870 (1,870/2,820, 66.3%) of them have a good understanding of its effect against seasonal influenza. The proportion of participants with awareness and knowledge was higher in the HCW group (93.3 and 72.3%, respectively) and health administrator group (97.1 and 85.7%, respectively) than that in the general population group (81.6 and 44.0%, respectively, *p*-values < 0.05). Respondents presented confidence in distinguishing flu from a regular cold (74.2%), which was less than their awareness of influenza vaccines (87.1%). Due to the extremely high awareness rate of the health administrator group, only influencing factors in general population group and HCW group were explored. As shown in Table 2, only in the general population group were males (aOR = 0.64, 95% CI: 0.50–0.83), ethnic minorities (aOR = 0.67, 95% CI: 0.47–0.97), and those with a degree lower than bachelor (aOR = 0.39, 95% CI: 0.24–0.63) had significantly lower awareness of influenza vaccines. Compared to those with self-reported family annual income under 50 thousand yuan (RMB), the awareness was significantly associated with income at the level of 60–100 k per year (aOR = 1.44, 95% CI: 1.08–1.93 in general population group; aOR = 3.06, 95% CI: 1.61–5.78 in HCW group), and 110–350 k per year (aOR = 1.43, 95% CI: 1.03–2.00 in general population group; aOR = 3.53, 95% CI: 1.72–7.31 in HCW group) (Table 2).

**Table 2:** Socio-demographic factors associated with awareness rate and knowledge rate of influenza vaccines among groups-multivariable logistic regression analysis.

**Characteristics**	**Awareness rate**				**Knowledge rate**					
	**General population (95% CI)**	***P*.value**	**Healthcare workers (95% CI)**	***P*.value**	**General population (95% CI)**	***P*.value**	**Healthcare workers (95% CI)**	***P*.value**	**Health administrator (95% CI)**	***P*.value**
Age, years										
18–25	Reference		Reference		Reference		Reference		Reference	–
26–35	0.91 (0.65–1.28)	0.59	1.30 (0.48–3.29)	0.59	0.84 (0.64–1.11)	0.22	0.90 (0.49–1.64)	0.74	1.07 (0.19–4.66)	0.93
36–45	1.18 (0.63–2.26)	0.62	1.59 (0.47–5.18)	0.45	1.20 (0.75–1.94)	0.44	0.97 (0.47–1.97)	0.93	1.18 (0.18–6.40)	0.86
>45	1.01 (0.54–1.90)	0.97	1.29 (0.37–4.41)	0.68	1.14 (0.70–1.85)	0.60	0.99 (0.46–2.07)	0.97	1.33 (0.20–7.30)	0.75
Gender										
Female	Reference		Reference		Reference		Reference		Reference	
Male	**0.64 (0.50–0.83)**	**<0.01**	0.65 (0.37–1.19)	0.15	**0.73 (0.60–0.89)**	**<0.01**	**0.66 (0.47–0.93)**	**0.018**	0.62 (0.33–1.15)	0.13
Ethnic										
Han	Reference		Reference		Reference		Reference		Reference	
Others	**0.67 (0.47–0.97)**	**0.03**	0.55 (0.29–1.11)	0.08	0.75 (0.54–1.03)	0.07	0.69 (0.45–1.06)	0.08	0.89 (0.31–3.28)	0.84
Educational background										
Master and above	Reference		Reference		Reference		Reference		Reference	
Bachelor or equal	0.68 (0.45–1.00)	0.06	1.11 (0.60–1.99)	0.72	0.79 (0.59–1.05)	0.10	0.92 (0.66–1.28)	0.63	1.36 (0.67–2.71)	0.39
Senior high or below	**0.39 (0.24–0.63)**	**<0.01**	0.3 (0.09–1.20)	0.06	**0.33 (0.22–0.49)**	**<0.01**	0.38 (0.14–1.04)	0.06	2.02 (0.48–9.95)	0.36
Marital statue										
Unmarried	Reference		Reference		Reference		Reference		Reference	
In marriage	1.45 (0.93–2.29)	0.11	1.25 (0.59–2.63)	0.56	**1.54 (1.09–2.18)**	0.02	1.45 (0.94–2.23)	0.09	0.84 (0.32–2.10)	0.70
Others	1.05 (0.47–2.54)	0.91	0.35 (0.11–1.29)	0.09	1.30 (0.61–2.70)	0.49	0.57 (0.24–1.38)	0.21	1.09 (0.13–23.99)	0.95
Area										
Urban	Reference		Reference		Reference		Reference		Reference	
Rural	0.9 (0.69–1.19)	0.46	1.26 (0.45–4.24)	0.68	**0.70 (0.56–0.88)**	**<0.01**	1.51 (0.75–3.15)	0.26	**0.27 (0.09–0.81)**	**0.017**
Annual household income, thousand yuan										
<50	Reference		Reference		Reference		Reference		Reference	
60–100	**1.44 (1.08–1.93)**	**0.01**	**3.06 (1.61–5.78)**	**<0.01**	1.05 (0.83–1.33)	0.67	**2.81 (1.85–4.28)**	**<0.01**	1.33 (0.50–3.35)	0.55
110–350	**1.43 (1.03–2.00)**	**0.03**	**3.53 (1.72–7.31)**	**<0.01**	1.24 (0.95–1.61)	0.11	**3.31 (2.12–5.20)**	**<0.01**	2.60 (0.95–6.81)	0.06
>350	1.27 (0.69–2.51)	0.47	4.45 (0.82–83.21)	0.16	1.12 (0.67–1.84)	0.67	**3.83 (1.41–12.31)**	**0.013**	5.00 (0.74–101.04)	0.16

Bolded text indicates statistically significant (*P*-values < 0.05).

Taking knowledge rate as a predictive factor, those with a degree lower than a bachelor's (aOR = 0.33, 95% CI: 0.22–0.49), and married individuals (aOR = 1.54, 95% CI: 1.09–2.18) had significantly changed only in the general population group. Males had less knowledge than females in the general population and HCW groups (aOR = 0.73, 95% CI: 0.60–0.89 in general population group; aOR = 0.66, 95% CI: 0.47–0.93 in HCW group), and rural residents knew less about influenza vaccines in the general population and health administrator groups (aOR = 0.70, 95% CI: 0.56–0.88 in general population group; aOR = 0.27, 95% CI: 0.09–0.81 in health administrator group). The more the HCW earned, the more likely they had acquired knowledge. More details are shown in Table 2.

### Willingness to be vaccinated against influenza

**Figure 3:**
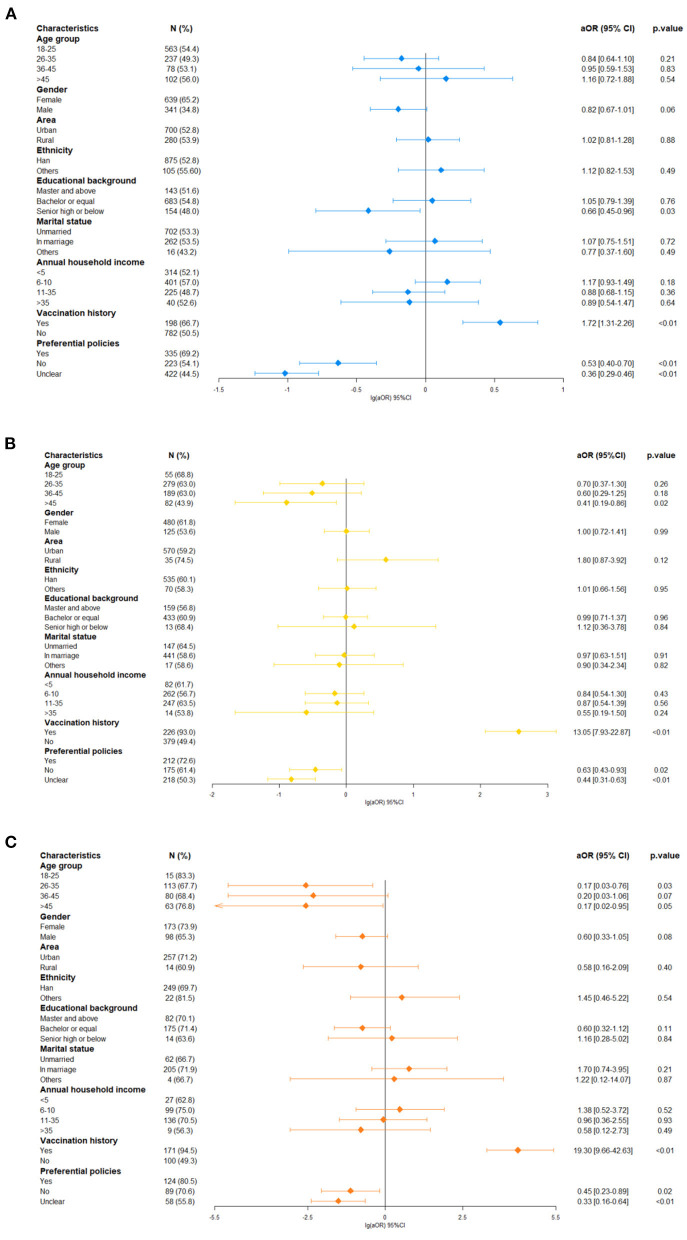
Factors associated with positive attitudes toward influenza vaccination. **(A)** In general population group, **(B)** in healthcare worker group, and **(C)** health administrator group.

The willingness to get influenza vaccines is difference among three group with the proportion of 53.1, 59.9, and 70.6% in general population group, HCW group and health administrator group separately (*p*-values < 0.05). The multivariate logistic regression of influenza vaccine awareness suggested that factors influencing people's willingness to get it were different among these groups. As shown in the Figure 3, low educational level (aOR = 0.66, 95% CI: 0.45–0.96), were associated with lower willingness to be vaccinated in general respondents (Figure 3A). For the HCW group, practitioners older than 45 years (aOR = 0.41, 95% CI: 0.19–0.86) were more reluctant to be vaccinated than those under 25 years (Figure 3B). For the health administrator group, compared to the young-aged group (18–25 years old), personnel aged 26–35 years (aOR = 0.17, 95% CI: 0.03–0.76), or 45 years and older (aOR = 0.17, 95% CI: 0.02–0.95) were less inclined to be vaccinated (Figure 3C). People who had received influenza vaccines in the past 5 years were more likely to accept it in future seasons, no matter in general population group (aOR = 1.72, 95% CI: 1.31–2.26), HCW group (aOR = 13.05, 95% CI: 7.71–22.10), or health administrator group (aOR = 19.30, 95% CI: 9.66–42.63). Considering favorable policies at residence, those out of related programs expressed reluctance to vaccination than those covered in three groups (aOR = 0.53, 95% CI: 0.40–0.70 in general population group; aOR = 0.63, 95% CI: 0.43–0.93 in HCW group; aOR = 0.45, 95% CI: 0.23–0.89 in health administrator group). Moreover, the same declarations were in respondents who did not know relevant policies (aOR = 0.36, 95% CI: 0.29–0.46 in general population group; aOR = 0.44, 95% CI: 0.31–0.63 in HCW group; aOR = 0.33, 95% CI: 0.16–0.64 in health administrator group).

### Attitude toward seasonal influenza vaccination recommendation in HCW

The HCW group was additionally asked about their attitude toward making a recommendation on influenza vaccination for others. A total of 70.8% (715/1,010) of the group, including 393 doctors and 322 nurses (55.0 vs. 45.0%, *p* = 0.27), expressed a firm willingness to give immunization recommendations (Table 3). Others (295/1,010) gave no precise indication of positive intention or a recommendation. Potential predictors of the likelihood of making recommendations on influenza vaccination were analyzed. As expected, staff gravitated toward recommendations after they got the recommendation (aOR = 2.13, 95% CI: 1.57–2.89). Doctors were more likely to make a recommendation than nurses (aOR = 0.72, 95% CI: 0.53–0.97). Two other factors were significant predictors, as shown in Table 3.

**Table 3:** Factors affecting healthcare workers' willingness to recommend influenza vaccines-univariable and multivariable logistic regression analysis.

**Factors**	**Total (*N*)**	**Willing (*N*)**	**Neutral or unwilling (*N*)**	**Willingness rate (%)**	**Univariable logistic regression**		**Multivariable logistic regression**	
Profession								
Doctor	544	393	151	55		Reference		Reference
Nurse	466	322	144	45	0.27	0.86 (0.65–1.13)	**0.03**	**0.72 (0.53–0.97)**
Have you ever been recommended to get a flu shot?								
Yes	609	480	129	67.1	**<0.01**	**2.63 (1.99–3.47)**	**<0.01**	**2.13 (1.57–2.89)**
No	401	235	166	32.9		Reference		Reference
Have you got influenza vaccines in the past 5 years?								
Yes	767	520	247	72.7	**<0.01**	**0.52 (0.37–0.74)**	0.46	0.86 (0.57–1.28)
No	243	195	48	27.3		Reference		Reference
Whether influenza vaccination service is provided at your unit?								
Yes	448	332	116	46.4		Reference		Reference
Not know	209	127	82	17.8	**<0.01**	**0.54 (0.38–0.77)**	0.40	0.84 (0.57–1.25)
No	353	256	97	35.8	0.61	0.92 (0.67–1.26)	0.20	0.80 (0.56–1.13)
Do you know that the government recommends vaccination for key groups?								
Yes	718	567	151	79.3	**<0.01**	**3.65 (2.73–4.89)**	**<0.01**	**2.22 (1.59–3.10)**
No	292	148	144	20.7		Reference		Reference
Do you know flu shots should be taken every year?								
Yes	677	538	139	75.2	**<0.01**	**3.41 (2.57–4.54)**	**<0.01**	**2.17 (1.55–3.05)**
No	333	177	156	24.8		Reference		Reference

Bolded text indicates statistically significant (*P*-values < 0.05).

### Perspectives of seasonal influenza vaccination promotion in health administrators

To promote influenza vaccine uptake, preventive health workers (373/384) had voted almost unanimously in favor of a vital catalytic role played by governments. The minority was reluctant mostly because the promotion of vaccination is beyond their duty (6/11). Community health service centers (77.3%) and Centers for Disease Control and Prevention (66.1%) were considered regular vaccination agencies. Among respondents, 216 (56.3%) supported the idea that the costs should be shared between the governments and citizens, while 67.2% thought governments should take charge of more than 50%, if not all, of expense allocation. Extensive publicity and education were selected as the way to promote flu vaccines by most administrators (90.6%). However, facing difficulties included insufficient budget and workforce (79.4%), followed by low public awareness (76.3%), and worries about the safety and effectiveness (52.6%) of the vaccine. Table 4 shows details to facilitate influenza vaccine adoption.

**Table 4:** Health administrators' opinions toward promoting influenza vaccines.

**Variable**	***N***	**%**
Do you agree that governments should take charge of influenza vaccination		
promotion? (*n* = 384)		
Yes, I agree.	373	97.1
No, I don't agree.	11	2.9
The acceptable way to pay for influenza vaccine is (*n* = 384)		
Self-funded	32	8.3
Self-funded 70–90%	15	3.9
Self-funded 50–70%	79	20.6
Self-funded 30–50%	101	26.3
Self-funded 10–30%	21	5.5
Fully covered by government.	136	35.4
What do you think the government should do to make the flu vaccine		
universal? (*n* = 373)		
Extensive publicity and education	338	90.6
To encourage vaccination for suitable and high-risk groups	331	88.7
Financial subsidies for suitable and high-risk groups	313	83.9
To introduce into immunization programs	269	72.1
Organized vaccination for suitable and high-risk groups	283	75.9
Vaccination agency could be (*n* = 384)		
Centers for disease control and prevention	254	66.1
Community health service centers, local clinics	297	77.3
Maternal and child care hospitals	137	35.7
General Hospitals	151	39.3
Schools	61	15.9
What difficulties do you think institutions at your level have in promoting		
universal access to vaccines? (*n* = 384)		
Insufficient budget and workforce.	305	79.4
Public awareness of vaccines against influenza is low.	293	76.3
Concerns about the safety and effectiveness of vaccines	202	52.6
Vaccination is not the most cost-effective way currently.	135	35.2
Vaccine supply chains are lagging behind.	173	45.1
Lack of scientifically feasible promotion plan.	114	29.7

These categories are not mutually exclusive.

For these questions, percentages do not total to 100% because each responder could have chosen more than one options.

## Discussion

This cross-sectional survey revealed a significant difference in influenza vaccination among groups that played different roles in health promotion. Our results support previous findings and add new insights. There was about a 30% difference between awareness of influenza vaccines and the intention to be vaccinated. Intentions do not always predict behaviors well. A further 35% decrease was observed in influenza vaccination records in the past 5 years. The annual vaccination rate should be even lower considering not every respondent take influenza vaccine every year. These influenza vaccination behavior patterns were slightly different among the three groups, but could be explained by some reasonable behavior and social drivers.

The influenza vaccine was widely recognized, but people were unaware of its benefits. Looking at the socio-demographic characteristics, the influencing factors of awareness status varied within gender, ethnicity, educational level, and annual family income, along with associated factors of knowledge in gender, educational level, marital status, residential area, and annual family income. It is almost universal that a broad group with main ethnic (ethnic-Han in China), higher educational background, and income resulted in greater chances to have health information and express respectable knowledge. Preferential policies impact influenza vaccination willingness, as other studies showed (21–23), illustrating that external incentives could be indispensable to all people. Interestingly, income as an economic factor was associated with awareness or knowledge but offset by other influencing factors in willingness. An assumption that may explain this is that economic conditions are relevant to literacy standards, but people may have a desire for health. Previous seasons' behavior predicted intentions well. Differences also existed among groups. Professions related to health usually require advanced degrees; thus, lower educational background is only significant in the general population group. Also, seniors in the health field with experience would become complacent about their health, which would not appear in the general population group.

Critical nodes of vaccination behavior detailed unexpected discrepancies in groups. The positive inclination toward influenza vaccination was not limited by the public's knowledge. Also, even a stronger desire to get vaccinated, they confessed, might attribute to a lack of health literacy, leading to their high expectation of vaccination or misunderstanding of the vaccine. Admittedly, health-related workers have a better command of this information, which contributes in part to willingness. However, the willingness generation mechanism of poor knowledge people is worth exploring, evaluating, and utilizing during influenza vaccination popularity. Further in-depth interviews will be conducive to figuring out how their illogical thinking and feelings had impact on their behavior.

A striking finding regarding to perception about influenza vaccination from the Wheelock et al. study held true in this survey (24). The public's misunderstanding of influenza vaccine against the virus was identified through the current survey, which was in accord with experienced administrators' concern about vaccine confusion. Even knowledge of flu vaccines in the HCW group was unsatisfactory. Those desirable means and reality impediments in Table 4, who, what, and how to deliver during health education should become a priority to weaken the unfavorable thinking and feeling. In the light of this survey, doctors and mass media were popular ways to disseminate influenza vaccine propaganda. Rural men who never went to colleges need to boost their health literacy. For unvaccinated people, knowledge and favorable policies predict their increased willingness. Effective communication should be concrete and heterogeneous (e.g., “Influenza vaccine can prevent young children from asthma or pneumonia caused by flu”) rather than generous description (e.g., “Influenza vaccines protect you”). Also, communications need to be forward (e.g., “Well, get vaccinated”) rather than an optional approach (e.g., “What do you want to do about it?”) (24, 25).

HCWs are part of vaccine willingness research. Their health conditions guarantee essential medical resources in any infectious disease emergency (26). For influenza vaccination, they are always given high priority for taking and offering recommendations to others, and thus their individual behavior and recommendations have formed a social process when interacting with others within their social network. A recommendation is one of the strongest factors in vaccination propensity, specifically by boosting propaganda and encouraging people to get it early. Vaccination records within 5 years in this study among respondents were higher than average (15%) but below the optimum level. Multivariance results showed that vaccination history affected the likelihood of being vaccinated but not giving advice, demonstrating that personal behavior does not influence a practitioner's recommendation (27). As a priority vaccinated group, their vaccination could prevent both themselves and patients from respiratory infections. Healthcare providers who have been advised are more likely to deliver vaccination recommendations to others in our survey, indicating another important role in which they play in facilitating the uptake rate. From this, on-site influenza vaccination could give practitioners policy support, namely enhancing the possibility of recommending it under an active atmosphere. It would also serve as a cue to perform the decision-making process for others.

In line with previous evidence (28, 29), vaccination history in former seasons is conducive to future seasons. This hints to administrative departments to take action before it is too late. Practical issues including vaccination expense, accessing vaccination services and relative service experience are direct antecedents influencing influenza vaccination. Due to the cost of influenza vaccines, diverse reimbursement strategies have been used across China. This study suggests that having access to favorable policies is the main factor affecting vaccination propensity. Hit by COVID-19 led to residents in Shanghai expressing an increased positive inclination toward influenza vaccines, but actions would be catalyzed only if vaccines were available at no cost (30). Policies of influenza vaccination should also take the interests of the young and middle-aged into account. Such programs may assist people in making healthy lifestyle choices. Almost all administrators agreed that governments play a vital role in promoting influenza vaccination. Since administrators indicated inadequate resources, they can emphasize HCWs' role in social network and work with them. Supportive environments learning from the successful COVID-19 vaccination campaign could be fostered through introducing mobile carts or on-site vaccination, providing paid time off for employees with side effects after receiving a vaccine, and sending personalized notifications of vaccine compliance status and scheduling options (31). Such workable programs produced the desired result though multi-sectors apportioned cost to reduce the practical barriers together.

Our study has practical implications for influenza vaccine promotion. First, factors that impact the young and middle-aged population's attitudes have been brought into focus. A gap still exists in awareness, willingness, and vaccination. Influencing factors of those who had differences also maintained close relationships. Awareness and knowledge are necessary but not the trigger to act (32, 33). Moreover, behavioral and social science research proposed miscellaneous theories to build the foundation of intervention strategies. Identifying factors at different levels in this study-intrapersonal, interpersonal, organizational, and governmental is essential to clear intervention function and develop programs and incorporate resources.

There are some limitations in this study. Primarily, the cross-sectional nature of the present work does not allow the comparison of changes in vaccination intention and behavior from time to time, but we took the previous 5 years' vaccination status in our survey and some of the similar purpose surveys as reference. It also indicates that such a survey could be given at regular intervals. Secondly, respondents chosen according to this study's purpose may have produced bias. Convenience sampling was more likely to lead to limited representative of our results. However, we included participants from all seven administrative regions in China with a relatively large sample size to minimize this limitation, and outcomes were compared between different groups. Also, the cost was not considered in the general population group, making us unable to do a willing-to-pay analysis.

## Conclusions

Vaccine hesitancy has posed a worldwide threat and impeded the progress of avoiding vaccine-preventable diseases including influenza. To close the influenza vaccination coverage gap, those with a willingness would be targeted in future vaccination campaigns, while those hesitant are even more in need of a regular monitor and strong intervention to stimulate their minds' change. Vaccine-seeking is a complex behavior. The double-edged role played by HCW was detailed in this study's results. The practical information provided by administrators may help advocacy campaigns in the coming seasons. Leveraging physicians and nurses as paradigms and informants could be considered as a top priority. Our findings inspire a deeper assessment of other contributing social factors and socio-psychological variables in seasonal influenza and other vaccines to better interpret the vaccination uptake behavior.

## Data availability statement

The data analyzed in this study is subject to the following licenses/restrictions: The datasets generated and/or analyzed during the current study are not publicly available due to confidentiality policies but are available from the corresponding author on reasonable request. Requests to access these datasets should be directed to MJ: jiamengmeng@cams.cn.

## Ethics statement

This study was reviewed and approved by the National Cancer Center/Cancer Hospital, Chinese Academy of Medical Sciences and Peking Union Medical College (approval number: 20/054-2250, approval date: March 12, 2020). Informed consent was obtained at the beginning of the survey, and those who failed to provide consent could not have access to the survey. Datasets were anonymous and prevented the identification of any individual study subject by the research team at any stage of the study.

## Author contributions

MJ, Y-lQ, and LF led the conception and design of the study. HY, ZS, and SL conducted the questionnaire. BJ and ZW were closely involved in data analysis and interpretation. BJ wrote the manuscript. MJ and LF participated in data interpretation and revised the manuscript critically for important intellectual content. All authors read and approved the final manuscript.

## Funding

This study was funded by Non-profit Central Research Institute Fund of Chinese Academy of Medical Sciences (CAMS) (2021-RC330-002), the CAMS Innovation Fund for Medical Sciences (2020-I2M-1-001), China Postdoctoral Science Foundation (2021T140068). The funders played no role in the design of the study and collection, analysis, and interpretation of data and in writing the manuscript.

## Conflict of interest

The authors declare that the research was conducted in the absence of any commercial or financial relationships that could be construed as a potential conflict of interest.

## Publisher's note

All claims expressed in this article are solely those of the authors and do not necessarily represent those of their affiliated organizations, or those of the publisher, the editors and the reviewers. Any product that may be evaluated in this article, or claim that may be made by its manufacturer, is not guaranteed or endorsed by the publisher.
